# A Mini Review on Borate Photocatalysts for Water Decomposition: Synthesis, Structure, and Further Challenges

**DOI:** 10.3390/molecules29071549

**Published:** 2024-03-29

**Authors:** Xiaorui Sun, Jia Yang

**Affiliations:** 1 Chongqing Key Laboratory of Inorganic Special Functional Materials, College of Chemistry and Chemical Engineering, Yangtze Normal University, Fuling, Chongqing 408100, China; sunxiaoruiyznu@163.com; 2 MOE Key Laboratory of New Processing Technology for Nonferrous Metals and Materials, College of Materials Science and Engineering, Guilin University of Technology, Guilin 541004, China; 3 Guangxi Key Laboratory of Optical and Electronic Materials and Devices, Guangxi Universities Key Laboratory of Nonferrous Metal Oxide Electronic Functional Materials and Devices, Guilin 541004, China

**Keywords:** borate, photocatalyst, water decomposition, synthetic chemistry, structural feature

## Abstract

The development of novel photocatalysts, both visible and UV-responsive, for water decomposition reactions is of great importance. Here we focused on the application of the borates as photocatalysts in water decomposition reactions, including water splitting reaction, hydrogen evolution half-reaction, and oxygen evolution half-reaction. In addition, the rates of photocatalytic hydrogen evolution and oxygen evolution by these borate photocatalysts in different water decomposition reactions were summarized. Further, the review summarized the synthetic chemistry and structural features of existing borate photocatalysts for water decomposition reactions. Synthetic chemistry mainly includes high-temperature solid-state method, sol-gel method, precipitation method, hydrothermal method, boric acid flux method, and high-pressure method. Next, we summarized the crystal structures of the borate photocatalysts, with a particular focus on the form of the B-O unit and metal-oxygen polyhedral in the borates, and used this to classify borate photocatalysts, which are rarely mentioned in the current photocatalysis literature. Finally, we analyzed the relationship between the structural features of the borate photocatalysts and photocatalytic performance to discuss the further challenges faced by the borate photocatalysts for water decomposition reactions.

## 1. Introduction

The importance of the carbon cycle is well-known, which is due to the many arguments made by scientific researchers and the publicity given to it by governments over the past few decades. The hydrogen cycle is important as well. Hydrogen has a very large number of applications, such as a direct clean energy source, hydrogen fuel cells, chemical feedstock, and so on [1,2]. The feedstocks from which hydrogen production is sourced are natural gas, water, coal, and biomass [3]. Of these, water is readily available and the cheapest of the important materials. Hydrogen production from water now has two important technological paths: electrolysis of water [4] and photolysis of water [5]. Due to the advantages of powder photocatalysts in hydrogen evolution by photocatalytic reaction, such as ease of handling and scale-up, we will next focus on the study of water decomposition by light irradiation.

In terms of what most photocatalytic researchers know about the photocatalytic decomposition of water, it is generally accepted that this line of research began with the Honda-Fujishima effect reported by two researchers at the University of Tokyo in 1972 [6,7]. Based on the effect, more and more semiconductors are being studied as photocatalysts, and as of now, the reported photocatalysts include organic polymers [8,9], inorganic compounds [10,11,12], and even organic-inorganic complexes [13,14]. From the periodic table of elements, these photocatalysts can be further classified as halates [15,16], oxides [17,18], sulfides [19,20], selenides [21,22], nitrides [23,24], phosphates [25,26], carbon materials [27,28], borates [11,29], tantalates [30,31], niobates [32,33], tungstates [34,35,36], etc. Among them, the borates are new photocatalysts that have emerged in the last decade or so and have attracted much attention because of their rich structures.

There is already a wide variety of natural borates, and the addition of synthetic borates has resulted in more than 3900 species of borates in existence [37]. Microstructurally, zero-, one-, two-, and three-dimensional structures are included in borates [11], suggesting that borates are structurally tunable. The ability of borates to become well-known fluorescent and nonlinear optical materials also benefits from these structural features [38,39,40,41]. With the rise of photocatalytic research, exploring the photocatalytic performances of existing borate materials has attracted the attention of many researchers.

In this review, we summarized all borate photocatalysts using for photocatalytic water decomposition reactions, including water splitting reaction, photocatalytic hydrogen evolution half-reaction, and photocatalytic oxygen evolution half-reaction, to give researchers a complete picture of the current state of research. The synthetic chemistry and structural features of borate photocatalysts are discussed in depth. Our insights into existing borate photocatalysts for water decomposition reactions are presented based on the structure-determined property perspective, and possible challenges and corresponding solutions are suggested.

## 2. Fundamentals of Borate Photocatalytic Water Decomposition Reactions

### 2.1. The Mechanism of the Water Decomposition Reaction of Borate Photocatalysts

**Figure 1:**
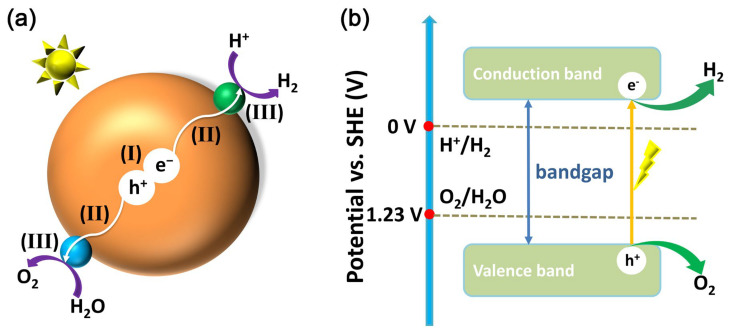
(**a**) Schematic illustration of water splitting over a borate photocatalyst: (I) light absorption and generation of photogenerated electron-hole pairs, (II) charge separation and migration to the surface of the co-catalyst, and (III) hydrogen and oxygen evolution reactions over the co-catalyst. (**b**) Schematic representation of the energy levels of water splitting subject to one-step excitation on a photocatalyst.

The basic principle of water splitting over borate photocatalysts is the same as that over other types of photocatalysts. As shown in Figure 1a, the large brown ball represents a borate photocatalyst, the small green ball represents a co-catalyst that assists in hydrogen evolution, and the small cyan ball represents a co-catalyst that assists in oxygen evolution. The photocatalytic water splitting reaction generally has three steps: (I) The first step is that the borate photocatalyst absorbs light and is excited by light to produce photogenerated electron-hole pairs. (II) The second step is the migration of photogenerated electron-hole pairs to the photocatalyst after separation; photogenerated electrons tend to accumulate on hydrogen evolution co-catalysts, while photogenerated holes tend to accumulate on oxygen evolution co-catalysts. (III) Photogenerated electrons and protons on a hydrogen evolution co-catalyst produce hydrogen, and photogenerated holes and water molecules on an oxygen evolution co-catalyst produce oxygen.

The equations of water splitting are as follows:

2H_2_O → 2H_2_ + O_2_ (1)

4H^+^ + 4e^−^ → 2H_2_ (2)

2H_2_O → O_2_ + 4H^+^ + 4e^−^ (3)

The relationship between the energy level structure of the photocatalyst and the redox electrode potential required for water decomposition can be seen in Figure 1b. The blue axes mark the two standard electrode potentials for redox required for water decomposition under ideal conditions, 0 V for hydrogen evolution and 1.23 V for oxygen evolution. Photocatalysts are generally semiconductors that possess a bandgap, which is the difference between the bottom potential of the conduction band (CB) and the top potential of the valence band (VB). When a photocatalyst is irradiated by light with an energy greater than the bandgap, photogenerated holes are generated in the valence band and photogenerated electrons are generated in the conduction band of the photocatalyst.

It is then necessary to match the potentials of the CB and VB with the electrode potentials required for water decomposition. The case shown in Figure 1b is that the potential of CB is more negative than that of hydrogen precipitation and the potential of VB is more positive than that of oxygen precipitation, which meets the potential requirements for total decomposition of water. Conversely, CB has an insufficient hydrogen evolution potential and can theoretically only undergo an oxygen evolution half-reaction; VB has an insufficient oxygen evolution potential and can theoretically only undergo a hydrogen evolution half-reaction.

### 2.2. Several Concepts of Photocatalytic Water Decomposition Reactions

To facilitate the description and discussion in the subsequent papers, a few important concepts are listed first in compliance with the more accepted viewpoints in photocatalysis research.

**Water Splitting Reaction:** In the strictest sense, water splitting refers to the continuous evolution of hydrogen and oxygen in the presence of light, with a molar ratio of hydrogen to oxygen of 2 to 1 (see Equation (1)) [42]. However, it is more lenient on the aqueous solution being decomposed, and can be water without impurity, or the pH of water can be adjusted with the addition of acids or bases.

**Hydrogen Evolution Half-Reaction (HEHR):** HEHR means that the aqueous solution contains a photogenerated hole sacrificer, such as methanol [43,44], triethanolamine [45,46], so the photogenerated electrons mainly react with protons in the aqueous solution to produce hydrogen (see Equation (2)).

**Oxygen Evolution Half-Reaction (OEHR):** OEHR is an aqueous solution that contains a photogenerated electron sacrificer, such as Ag^+^ [47,48], Na_2_S_2_O_8_ [49], so the photogenerated holes react mainly with water molecules in the aqueous solution to produce oxygen (see Equation (3)).

**Hydrogen/Oxygen Evolution Rate (HER or OER):** The rate of hydrogen or oxygen evolution obtained from photocatalytic water decomposition experiments is usually given in units of μmol/h, but to facilitate comparisons between the works of different groups the rate unit of μmol/h/g is used.

## 3. Synthetic Chemistry of Borate Photocatalysts

The effect of the synthetic method of the photocatalysts on the photocatalytic performance is significant, which is mainly attributed to the large differences in the specific surface area, morphology, and defects of the samples obtained by different synthesis methods. Therefore, in this section, we summarise the synthetic methods that have been reported up to now for the preparation of borate photocatalysts.

### 3.1. High-Temperature Solid-State Method

The traditional high-temperature solid-state method or solid-state method uses direct mixing of solid raw materials, grinding, and then direct calcining to obtain the target sample. The term “high temperature” is used in relation to the synthesis temperature of other soft chemical synthesis methods. The synthesis of borates by high-temperature solid-state method is relatively simple and effective, for example, K_3_Ta_3_B_2_O_12_ [50,51], InBO_3_ [52], Ga_4_B_2_O_9_ [53], Cd_12_Ge_17_B_8_O_58_ [54], CuB_2_O_4_ [55], Cu_3_B_2_O_6_ [55], PbGaBO_4_ [56], and Ni_a_Co_3−a_O_4_ [49], can be prepared by this method. However, only thermodynamically stable phases can be obtained by this method, and kinetically stable phases cannot be obtained. In addition, some of the raw materials are prone to decompose at high temperatures and deviate from the target stoichiometric ratios, which is also a disadvantage of this method for the synthesis of borates.

It is worth mentioning that Yang T. et al. did not use gallium oxide directly but activated gallium oxide with nitric acid in the synthesis of Ga_4_B_2_O_9_ by high-temperature solid-state method, and this operation led to the superior photocatalytic performance of the sample obtained by high-temperature solid-state method than those obtained by hydrothermal method and sol-gel method in this research system [53]. In addition, they used this improved high-temperature solid-phase method again in their subsequent study of the photocatalytic properties of PbGaBO_4_ and named it the solution-assist solid-phase method [56]. The hydrogen evolution performance of the PbGaBO_4_ samples obtained by the solution-assist solid-state method without the addition of co-catalysts is slightly higher compared to that of the PbGaBO_4_ samples obtained by the conventional solid-state method.

### 3.2. Complex Gelation Method or Sol-Gel Method

This method was mentioned in the preparation of K_3_Ta_3_B_2_O_12_ by Kudo A. et al. [57] The central step in the synthesis procedure, apart from the operation of removing the chloride ions, is the preparation of a dry gel, and then after calcination, to obtain the target sample. The main reagents of the method were soluble tantalum salt, potassium nitrate, and boric acid, in addition to a large amount of citric acid as a complexing agent. In fact, this method is generally known as the sol-gel method in the synthesis of Ga_4_B_2_O_9_ reported by Yang T. et al. [53]. The raw materials included homemade soluble gallium nitrate, boric acid, and citric acid, this was followed by the preparation of dry gel and finally calcination to obtain the sample Ga_4_B_2_O_9_.

Later, Matsumoto Y. et al. made some improvements to this method when they revisited the synthesis of K_3_Ta_3_B_2_O_12_ [58]. For example, the substitution of polyvinyl alcohol for citrate is known as the PVA method, and the additional addition of propylene glycol to the original reagents is known as the polymerizable complex method. The use of these modifications requires attention to the conditions of synthesis, such as the ratio of raw materials, the temperature of synthesis, and the time of synthesis, otherwise, impurities are obtained during the synthesis of the K_3_Ta_3_B_2_O_12_ sample.

### 3.3. Precipitation Method

In their first report on the decomposition of water by InBO_3_ as a photocatalyst, Kudo A. et al. synthesized the sample using a conventional solid-state method and also used a two-step precipitation method to prepare the sample as a control [52]. The specific steps are as follows: in the first step, indium nitrate and excess boric acid as the initial raw materials are first dissolved in hot water, and then ammonia solution is added till the pH value of the solution is 7, at which time the In^3+^ ions in the solution are precipitated as In(OH)_3_. In the second step, the precipitate-containing solution is cooled with ice water which will allow the boric acid to recrystallise again, and the precipitate is then separated to obtain the precipitate and dried at 333 K for one day in air. After drying, the precursor is calcined at 823–1273 K for 0.5–10 h to obtain the target sample. It is worth mentioning that this method is 200–300 °C lower than the synthesis temperature using high-temperature solid-phase methods and the calcination time is more than halved.

**Figure 2:**
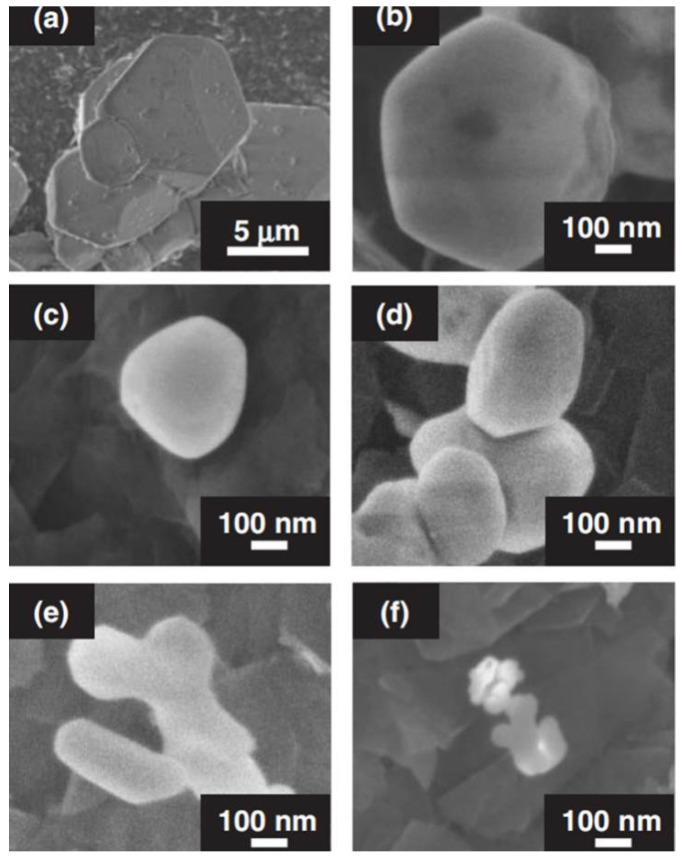
Scanning electron microscope (SEM) images of InBO_3_ prepared by (**a**) solid-state reaction and (**b**–**f**) precipitation. Preparation conditions: (**a**) 1523 K for 72 h, (**b**) 1273, (**c**) 1073, (**e**) 1023, and (**f**) 973 K for 2 h, a 10-fold excess of H3BO_3_ was used in the starting material and (**d**) 1023 K for 2 h, a 20-fold excess of H3BO_3_ was used in the starting material [52].

Figure 2 shows the SEM of InBO_3_ prepared by solid-state reaction and precipitation methods under different conditions. The particle size of InBO_3_ prepared by solid-state reaction is 5–10 μm, while the particle size of InBO_3_ prepared by precipitation method is 50–200 nm, which becomes smaller as the preparation temperature decreases. In particular, small particles of 50 nm were obtained by calcination at 973 K (see Figure 2f). It is also seen that the more boric acid added to the initial reagent, the larger the particles of InBO_3_ obtained (see Figure 2d,e).

### 3.4. Hydrothermal Method

**Figure 3:**
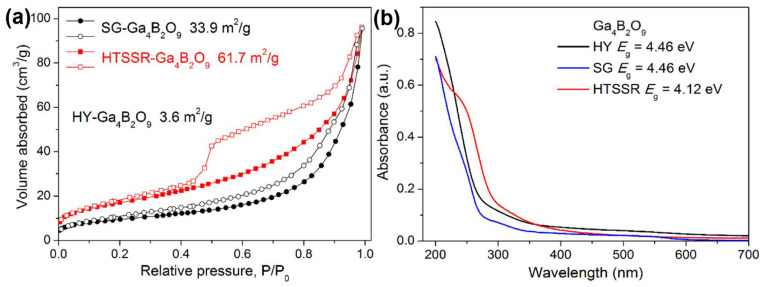
(**a**) N_2_ adsorption-desorption curves at 77 K. The filled and open symbols represent the adsorption and desorption branches, respectively. The specific surface areas are estimated by the BET method. (**b**) UV–vis reflectance spectra for various Ga_4_B_2_O_9_ samples [53].

The hydrothermal method was also used for the preparation of Ga_4_B_2_O_9_ by Yang T. et al. [53] The steps are simple: homemade gallium nitrate and boric acid are used as raw materials, with water as the medium, and then sealed in a polytetrafluoroethylene (PTFE) liner, sleeved with a stainless steel reactor, and reacted at 240 °C for 5 days, and finally a needle-like single crystal sample is obtained. Yang T. et al. used modified solid-state, sol-gel, and hydrothermal methods to synthesize Ga_4_B_2_O_9_, of which the samples obtained by the hydrothermal method had the worst photocatalytic decomposition of water, and the best photocatalytic decomposition of water was obtained from the sample obtained by the solid-state method. In this system, the samples obtained by the hydrothermal method have a larger bandgap and have the smallest specific surface area of 3.6 m^2^/g (see Figure 3).

Jia Z. et al. reported the preparation of two new copper-based borate photocatalysts with cubic supramolecular cages by a hydrothermal method: H_2_Na_2_K_2_[(μ4-O)Cu_4_@B_20_O_32_(OH)_8_]∙21H_2_O (denoted as Cu-borate-1) and H_2_Rb_1__.6_K_2.4_[(μ4-O)Cu_4_@B_2__0_O_32_(OH)_8_]∙15H_2_O (denoted as Cu-borate-2) [59].

Gu Q. and Liu Z. et al. collaborated to report an ultrathin two-dimensional borate anions B_4_O_5_(OH)^2−^ intercalated layered double hydroxide (ZnGa-BLDH) nanosheets with a thickness of 5–6 nm (2–3 layers stacked) for the dye-sensitized photocatalytic coupled reaction of hydrogen production with pollutant degradation [60].

### 3.5. Boric-Acid Flux Method

Similar to the hydrothermal method, a PTFE-lined stainless steel hydrothermal reactor is used, except that water is no longer added to the reactor, but only boric acid in a molten state is used as a solvent. For example, Yang T et al. sealed a small amount of homemade gallium nitrate and far too much boric acid (3 g) together in a hydrothermal reactor, and then 220 °C to maintain 5 days to obtain Ga_9_B_18_O_33_(OH)15∙H_3_B_3_O_6_∙H_3_BO_3_ (denoted as Ga-PKU1) sample [61,62]. Subsequently, they synthesized In_1−x_Ga_x_BO_3_ samples by adding appropriate amounts of oxalic acid based on this method [63].

### 3.6. High-Pressure Method

The high-pressure method is significantly different from all of the above preparation methods in that it requires special equipment that can provide high temperature and high pressure which is generally more than 10 GPa according to the synthesis conditions of borate photocatalysts reported by Huppertz H. et al. [64,65,66]. For example, sample In_19_B_34_O_74_(OH)11 was synthesized at 1150 °C and 13 GPa [64], samples M_5_B_12_O_25_(OH) (M = In, Ga) at 1450 °C and 12 GPa [66], and sample Ga_2_B_3_O_7_(OH) at 700 °C and 10.5 GPa [65].

We summarize the comparative areas measured and particle sizes observed by scanning electron microscopy for borate photocatalysts prepared by different synthetic methods (see Table 1). Although four borate photocatalysts were obtained using the high-pressure method, information on their morphology and particle size is missing; based on their lower hydrogen production performance, it is reasonable to assume that they have a larger particle size or a smaller comparative area. The most borate photocatalysts were obtained using the solid-state method, and the comparative areas of the other photocatalysts did not even exceed 3 m^2^/g, except for Ga_4_B_2_O_9_, which was obtained using the modified solid-state method. Therefore, synthetic methods for preparing borate photocatalysts other than these two are more desirable. It is noteworthy that the samples obtained hydrothermally in the study of Ga_4_B_2_O_9_ had the smallest specific surface area, which is attributed to the fact that borates are more likely to grow into crystals with larger particle sizes under suitable hydrothermal conditions.

## 4. Structural Characterization of Borate Photocatalyst

From a crystallographic point of view, it is inconclusive which crystalline system or which space group of materials has better photocatalytic performances. The borates are structurally very diverse materials due to the combination of BO_3_ triangles and BO_4_ tetrahedra. Structural information on borate photocatalysts capable of decomposing water or methanol to produce hydrogen is summarised in Table 2.

In terms of crystal system, no borates with a cubic crystal system have been reported as photocatalysts for hydrogen evolution. Further analysis of the B-O units in these borate photocatalysts show that except for BO_4_ in CuB_2_O_4_, which is distributed in a three-dimensional network, the B-O units in the other borates are relatively isolated [67]. All the B-O units in the borate photocatalyst include BO_3_, BO_2_(OH), BO_4_, B_2_O_7_, B_2_O_4_(OH), B_3_O_6_, B_4_O_10_, B_4_O_7_(OH)_2_, B_4_O_5_(OH)_4_, and B_12_O_25_(OH) [50,51,52,53,54,55,56,57,58,59,60,61,62,63,64,65,66,67].

Of these B-O units, B_12_O_25_(OH) occurs only in In_5_B_12_O_25_(OH) and Ga_2_B_3_O_7_(OH) photocatalysts obtained using the high-pressure method, which has a very complex structure [65,66]. Another photocatalyst obtained by the high-pressure method is In_19_B_34_O_74_(OH)_11_, whose B-O unit is B_4_O_10_ [64]. The solution for hydrogen evolution of these three high-pressure method-prepared photocatalysts is pure methanol, which is special compared to other photocatalysts. Huppertz H. et al. have synthesized several borates by high-pressure methods, of which Ga_5_B_12_O_25_(OH) is structurally identical to In_5_B_12_O_25_(OH), however, its photocatalytic performance of hydrogen evolution has not been tested [66]. Therefore, this seems to imply that the high-pressure method is not the most suitable method for preparing borate photocatalysts, but it is still worth trying in developing new borate photocatalysts. Except for B_12_O_25_(OH), the number of B in the remaining B-O units ranges from 1 to 4. This indicates a principle for screening hydrogen precipitation photocatalysts from a large number of borate materials.

Furthermore, considering the cations in the borate photocatalysts, we note that the first transition metal borates have small bandgaps (≤3.3 eV), such as CuB_2_O_4_ [55], Cu_3_B_2_O_6_ [55], Co_3_B_2_O_6_ [49], Co_2_NiB_2_O_6_ [49], CoNi_2_B_2_O_6_ [49], Ni_3_B_2_O_6_ [49], Cu-borate-1 [59], and Cu-borate-2, [49] most of which are capable of decomposing water under visible light or simulated sunlight. Others include p-block borates, and both the second and third transition metal borates have bandgaps greater than 4.0 eV. Since the development of visible light-responsive photocatalysts is an important goal in photocatalytic research, this provides a second principle for the study of borate photocatalysts, which is that it is somewhat more likely to obtain narrow-bandgap borates by possessing the first transition metal element in the cationic composition of the borate.

**Table 2:** A summary of structural information on borate photocatalysts capable of decomposing water or methanol to produce hydrogen.

Photocatalyst	Crystal Structure	Crystal System	Spaces Group	B-O Unit	Comment	Bandgap /eV	Refs.
K_3_Ta_3_B_2_O_12_	tungsten bronze	hexagonal	P-62m	BO_3_ isolated	Corner-sharing triads of TaO_6_ octahedra are stacked along the c-axis and are connected by planar BO_3_ groups.	4.2	[50]
Cd_12_Ge_17_B_8_O_58_	-	tetragonal	P-4	B_2_O_7_ isolated	The [Ge_4_O_10.5_]n polyhedra-chains along the c-axis were interconnected by [Ge(B_2_O_7_)_4_]^28−^ clusters and the remaining vacancies were filled by Cd^2+^ to satisfy the charge neutrality.	4.27	[54,68]
PbGaBO_4_	-	orthorhombic	Pnma	BO_3_ isolated	These GaO_6_ chains are interconnected by rigid BO_3_ triangles, leaving Pb^2+^ in an asymmetric coordination.	4.10	[56]
ZnGa-BLDH	-	hexagonal	-	B_4_O_5_(OH)_4_^2−^	2- It obtained the borate anions B_4_O_5_(OH)_2_ intercalated ZnGa-BLDH ultrathin 2D nanosheets by a one-step hydrothermal method.	4.94	[60]
InBO_3_	calcite	trigonal	R-3cH	BO_3_ isolated	The InO_6_ octahedral units connect with each other by corner sharing, and link with the BO_3_ triangular units.	5.4	[62,63]
In_5_B_12_O_25_(OH)	-	tetragonal	I41/acd	B_12_O_25_(OH)	A set of twelve corner-sharing BO_4_ tetrahedra forms either a cuboctahedral cage or two six-membered curved strings that enlace the distorted In_2_O_6_ octahedra.	-	[66]
In_19_B_34_O_74_(OH)_11_		trigonal	R-3	B_4_O_10_	The central structural motifs in In_19_B_34_O_74_(OH)_11_ are [B_4_O_10_]^8−^ supertetrahedra located in the center of a -3 axis.	-	[64]
Ga_4_B_2_O_9_	mullite	monoclinic	C2/m	BO_3_ isolated BO_4_ isolated	The GaO_6_ octahedra share edges to form chains along the b-axis, and the chains are cross-linked by GaO_5_, BO_3_, and BO_4_ groups.	4.12	[53,69]
Ga_2_B_3_O_7_(OH)	-	orthorhombic	Cmce	B_12_O_25_(OH)	Similar to In_5_B_12_O_25_(OH).	4.0	[65]
Ga-PKU-1	-	trigonal	R3	BO_2_(OH) isolated B_2_O_4_(OH) isolated	Borate groups, in the form of BO_2_(OH) and B_2_O_4_(OH) fragments, attach to the GaO_6_ framework and neutralize the negative charges by sharing vertex oxygen atoms.	4.8	[61,70]
Cu-Borate-1	-	tetragonal	I-4	B_4_O_7_(OH)_2_ isolated	The {B_20_} cluster was built by four alternated [B_4_O_7_(OH)_2_] clusters and four BO_3_ triangles via common oxygen atoms.	2.3	[59]
Cu-Borate-2	-	tetragonal	I-4	B_4_O_7_(OH)_2_ isolated	The same as Cu-Borate-_1_.	2.3	[59]
CuB_2_O_4_	-	tetragonal	I-42d	B_3_O_6_ network	The CuO_4_ is at the centre of the quadratic axis and it is surrounded by a mesh of eight BO_4_ tetrahedra.	3.30	[55]
Cu_3_B_2_O_6_	-	triclinic	P-1	BO_4_ isolated	The layers are linked to one another by BO_4_ tetrahedra.	2.47	[55]
Co_3_B_2_O_6_	kotoite	orthorhombic	Pnmn	BO_3_ isolated	Each metal site is surrounded by an oxygen atom of planar borate groups to afford an octahedral environment.	-	[49]
Co_2_NiB_2_O_6_	kotoite	orthorhombic	Pnmn	BO_3_ isolated	The same as Co_3_B_2_O_6_.	-	[49]
CoNi_2_B_2_O_6_	kotoite	orthorhombic	Pnmn	BO_3_ isolated	The same as Co_3_B_2_O_6_.	-	[49]
Ni_3_B_2_O_6_	kotoite	orthorhombic	Pnmn	BO_3_ isolated	The same as Co_3_B_2_O_6_.	-	[49]

## 5. Borate Photocatalyst for Photocatalytic Decomposition of Water

### 5.1. Borate Photocatalyst for Overall Water Splitting

Among the water decomposition reactions, the water splitting reaction has been the most interesting and challenging one. Up to now, only K_3_Ta_3_B_2_O_12_ [50,51,57,58], InBO_3_ [52], Ga_4_B_2_O_9_ [53], Ga-PKU-1 [62], and Cd_12_Ge_17_B_8_O_58_ [54] have been reported to be capable of water splitting (see Table 3). From the polyhedral structure of the metal-oxygen in borate photocatalysts, it can be divided into one-dimensional chains and three-dimensional networks. Although these samples are only responsive to ultraviolet light, they show that borates can be used in water splitting reactions.

#### 5.1.1. One-Dimensional Chain Borate Photocatalyst

##### K_3_Ta_3_B_2_O_12_

K_3_Ta_3_B_2_O_12_ is the first photocatalyst among borates to be used in water decomposition reactions, and it was first reported by Kudo A. et al. in 2006 [50,51]. As of now, there are four papers on K_3_Ta_3_B_2_O_12_ and Kudo A. is one of the authors of three of them. Therefore, the results in these different papers should be more comparable.

**Figure 4:**
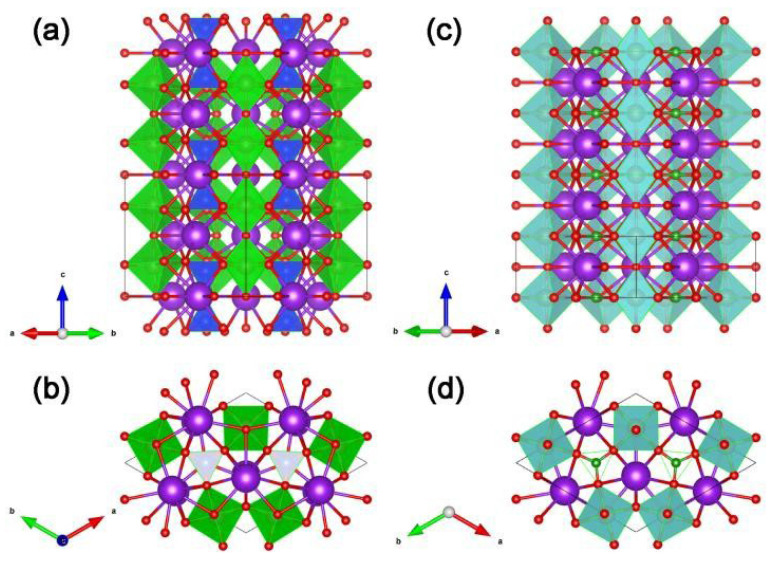
Structure views of K_3_Ta_3_Si_2_O_13_ along the (**a**) [110] direction and (**b**) c-axis. TaO_6_ is present as the green octahedron. SiO_4_ is present as the blue tetrahedron. Structure views of K_3_Ta_3_B_2_O_12_ along the (**c**) [110] direction and (**d**) c-axis. TaO_6_ is present as the cyan octahedron. BO_3_ is present as the green triangular. In addition, red and purple spheres represent O and K, respectively.

Since Kudo A. et al. have studied the photocatalytic hydrogen evolution of sample K_3_Ta_3_Si_2_O_13_ [71] and recognized that the crystal structures of samples K_3_Ta_3_Si_2_O_13_ and K_3_Ta_3_B_2_O_12_ are highly similar (see Figure 4), so they have interest to study the photocatalytic performance of K_3_Ta_3_B_2_O_12_. For example, in sample K_3_Ta_3_Si_2_O_13_, the TaO_6_ octahedra corners share with each other providing straight pillars parallel to the c-axis of the crystal (see Figure 4a), the TaO_6_ pillars are linked by SiO_4_ terahedral units (see Figure 4b). However, the SiO_4_ unit replaced by the BO_3_ unit is the crystal structure of K_3_Ta_3_B_2_O_12_ (see Figure 4c,d) [50,51]. It is worth mentioning that Kudo A. et al. argued that the chain-like structure of TaO_6_ octahedra in the crystal structure facilitates the transfer of photogenerated electrons and holes [51], and this idea has influenced the subsequent work of other researchers in developing borate photocatalysts, e.g., Yang T. et al. emphasized the role of the GaO_6_ chains for the photogenerated carriers in their work on PbGaBO_4_ [56].

Four methods, the solid-state method, complex gelation method, PVA method, and polymerizable complex method, are currently used to prepare K_3_Ta_3_B_2_O_12_ to probe its photocatalytic performance of water splitting [51,57,58]. It has a bandgap of around 4.1 eV, and it is also established that the bandgap of the sample obtained by the complex gelation method is 0.1 eV smaller than the bandgap obtained by the solid-state method. The photocatalytic performance of water decomposition for the K_3_Ta_3_B_2_O_12_ sample obtained from different synthesis conditions with and without NiO co-catalyst are given in Table 2. From the results, it can be concluded that K_3_Ta_3_B_2_O_12_ can decompose water without a co-catalyst, the NiO co-catalyst can effectively enhance the photocatalytic performance, and the sample obtained by using the complex gelation method has better photocatalytic performance of water splitting. The highest HER of the K_3_Ta_3_B_2_O_12_ sample is obtained by the complex gelation method, and the value is 5333.3 μmol/h/g. However, the K_3_Ta_3_B_2_O_12_ obtained by Matsumoto Y. et al. using the solid-state method did not repeat the photocatalytic performance of the K_3_Ta_3_B_2_O_12_ sample obtained by Kudo A. et al. using the same synthesis conditions, which Matsumoto Y. et al. interpreted as a decrease in the photocatalytic performance due to B defects created during the synthesis process that became the recombination center of the photogenerated electron-hole pairs [58].

##### Cd_12_Ge_17_B_8_O_58_

**Figure 5:**
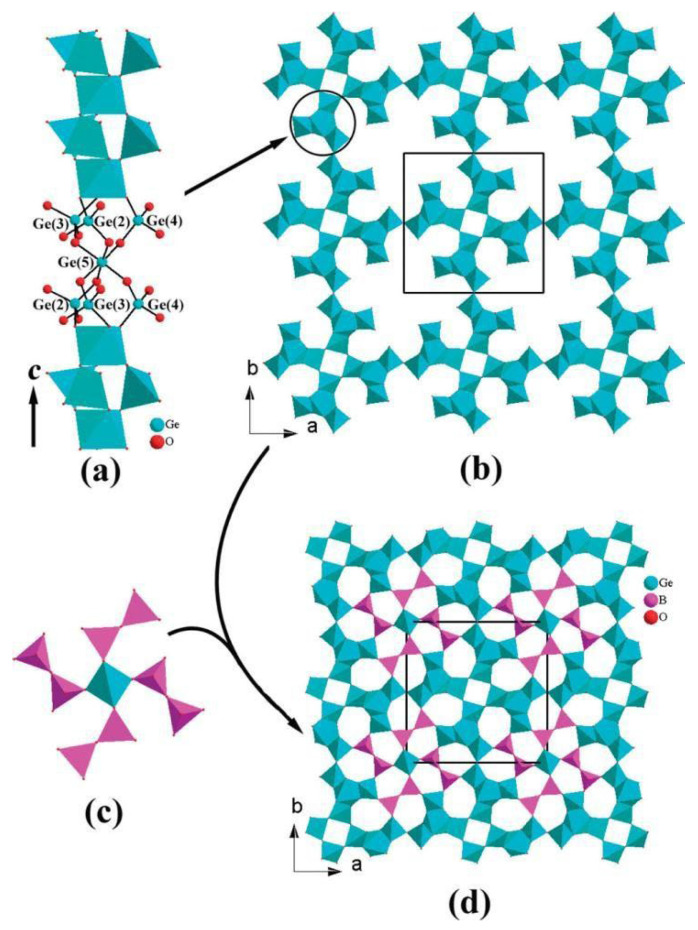
Scheme showing a 1-dimensional [Ge_4_O_12_]n chain (**a**) and construction of a 3-dimensional [Ge_4_O_10.5_]n network (**b**) and then a Ge(B_2_O_7_)4 unit (**c**) and construction of a 3-dimensional [Ge_17_B_8_O_58_]^24−^ anionic network (**d**) in Cd_12_Ge_17_B_8_O_58_ [68].

In Cd_12_Ge_17_B_8_O_58_, neighboring one-dimensional [Ge_4_O_12_]n chains are interconnected into a [Ge_4_O_10.5_]n open framework via corner sharing with large pores filled by big [Ge(B_2_O_7_)4]^28−^ clusters, leading to formation of three types of one-dimensional tunnels of 5-, 6-, and 7-membered rings along the c-axis which are occupied by the Cd^2+^ cations (see Figure 5) [68].

Yang T. et al. prepared a Cd_12_Ge_14_B_8_O_58_ sample by solid-state method and investigated its performance in the photocatalytic decomposition of pure water [54]. The enhancement of photocatalytic performance by Ag, Au, Pt, Pd, RuO_x_, CuO_x_, and NiO_x_ was systematically investigated, with the highest HER obtained on the NiO_x_/Cd_12_Ge_14_B_8_O_58_ sample. The HER of the NiO_x_/Cd_12_Ge_14_B_8_O_58_ sample is 163 μmol/h/g, which is 8.2 times of the HER of the Cd_12_Ge_14_B_8_O_58_ sample. 

#### 5.1.2. Three-Dimensional Network Borate Photocatalyst

##### InBO_3_

**Figure 6:**
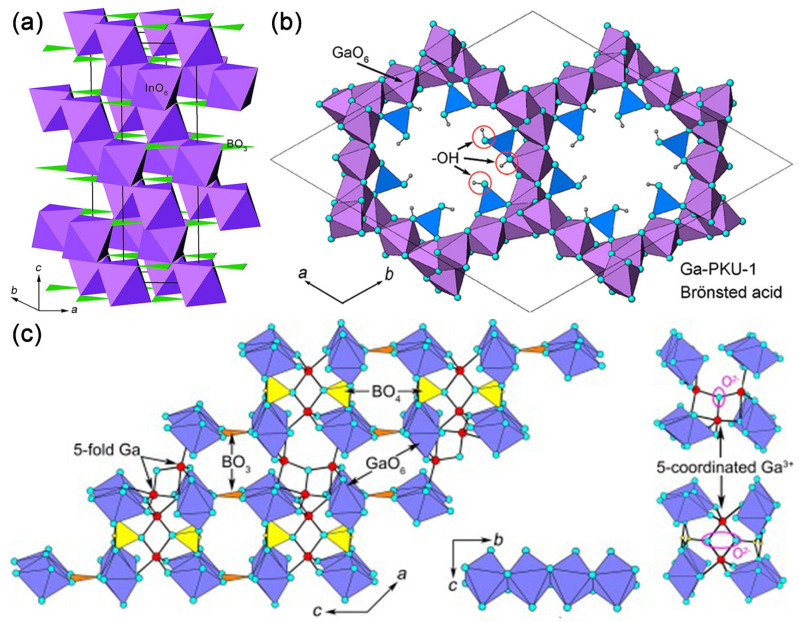
(**a**) A schematic view of the crystal structure of InBO_3_ in *R-3c*. InO_6_ and BO_3_ species are depicted as purple octahedra and green triangles [63]. (**b**) Structure view of Ga-PKU-1 along the c-axis [70] (**c**) Structure illustration of Ga_4_B_2_O_9_ with unsaturated coordination along the b-axis, octahedral chains along the a-axis, and μ3-O atoms linked to five-coordinated Ga^3+^ [72].

Later in 2010, Kudo A et al. concluded that InBO_3_ has a similar structure to K_3_Ta_2_B_2_O_12_ [52], so they investigated the photocatalytic water splitting of InBO_3_. The InBO_3_ possesses a calcite-type structure, where InO_6_ octahedra form a three-dimensional framework by sharing corners, leaving BO_3_ as interconnection groups (see Figure 6a) [63]. Kudo A. et al. systematically investigated the photocatalytic water splitting by preparing InBO_3_ using solid-state and precipitation methods, and then loading co-catalysts on InBO_3_ using photodeposition and impregnation methods.

The photocatalytic performance of InBO_3_ obtained using the precipitation method is much higher than that of the solid-phase method. However, without a co-catalyst, InBO_3_ cannot achieve a strict water splitting reaction, i.e., the molar ratio of hydrogen to oxygen is not 2. The loading of Ni, Au, Pd, Ru, and IrO_2_ on InBO_3_ by photodeposition, and the loading of RuO_2_, NiO, and NiO_x_ on InBO_3_ by impregnation showed that only NiO/InBO_3_ could achieve a rigorous water splitting reaction. The HER and OER of NiO/InBO_3_ are 796 and 404 μmol/h/g, respectively.

##### Ga-PKU-1

The framework of Ga-PKU-1 consists of GaO_6_ octahedra exclusively edge-sharing, forming 18- GaO_6_-membered structural channels along the c-axis. Borate groups, in the form of BO_2_(OH) and B_2_O_4_(OH) fragments, attach to the GaO_6_ framework and neutralize the negative charges by sharing vertex oxygen atoms (see Figure 6b) [61]. In addition, boric and metaboric acids are extraframework species, which are in the channel of the 18-GaO_6_ ring.

The Ga-PKU-1 was prepared by a boric-acid flux method. First, β-Ga_2_O_3_ was dissolved in concentrated HNO_3_ at 180 °C for 10 h in a closed system. The resultant solution evaporates to nearly dry by just opening to air. Thereafter, H_3_BO_3_ (3.0 g) was charged and the system was sealed again and maintained at 220 °C for another 5 days. Finally, the white powder product was washed with deionized water several times [62]. The effects of different co-catalysts on the photocatalytic performance of Ga-PKU-1 were systematically investigated, and finally, Ga-PKU-1 achieved photocatalytic water splitting in the presence of 1wt% RuO_x_ and 1wt% Pt dual co-catalysts. The HER and OER of the 1wt%RuOx-1wt%Pt-Ga-PKU-1 are 28.4 and 14.5 μmol/h/g, respectively.

##### Ga_4_B_2_O_9_

Ga4B2O9, in which GaO_6_ octahedra share edges in a trans-manner forming one-dimensional chains along the b direction, and the chains are further cross-linked by GaO_5_, BO_3_, and BO_4_ groups into a three-dimensional mullite-type structure (see Figure 6c) [69]. Yang T. et al. recognized that the three-dimensional connectivity of Ga−O polyhedra provides pathways for the migration of photogenerated carriers [53].

Ga_4_B_2_O_9_ was prepared by hydrothermal, sol-gel hair, and modified solid-phase methods, and their corresponding morphologies were micrometer single crystals, severely agglomerated nanorods, and straight nanostrips of hundreds of nanometers in length and <20 nm in diameter, respectively. The bandgap of the Ga_4_B_2_O_9_ sample obtained using the modified solid-state method is the smallest (4.12 eV), which is 0.35 eV smaller than the bandgaps of the samples obtained by the other two methods. Combined considering the morphology and bandgap of these samples, this explains that the sample obtained by the solid-state method has the best photocatalytic performance. The HER and OER from Ga_4_B_2_O_9_ obtained by the solid-state method for the decomposition of water in the absence of a co-catalyst were 118 and 47 μmol/h/g, respectively.

### 5.2. Borate Photocatalysts with Wide Bandgap (≥4.0 eV) for HEHR or OEHR

The study of borate photocatalysts that can be used for HEHR or OEHR is still necessary in the absence of a sufficient variety of borates that can be used for hydrolysis reactions. In this section, we classify the metal-oxygen formed polyhedra in borate photocatalysts into three categories based on their structure: one-dimensional chain, two-dimensional layer, and three-dimensional network. In Table 4, the sacrificial agents for the HEHR are: methanol solution, triethanolamine (TEOA) solution, and pure methanol. The sacrificial agents for the OEHR are AgNO_3_ solution and Na_2_S_2_O_8_ solution.

#### 5.2.1. One-Dimensional Chain Borate Photocatalyst

**Figure 7:**
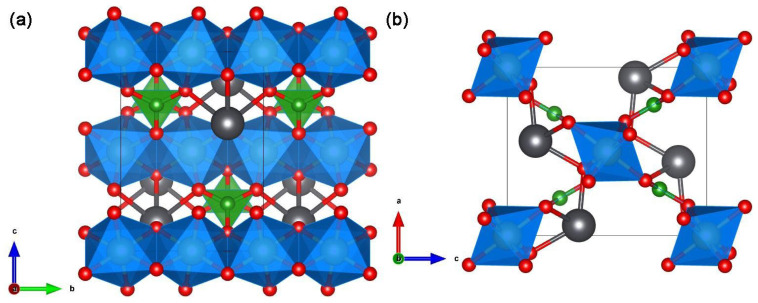
Structure views of PbGaBO_4_ along the (**a**) a-axis and (**b**) c-axis. GaO_6_ is present as the blue octahedron. BO_3_ is present as the green triangular. Red and grey spheres represent O and Pb, respectively.

PbGaBO_4_ comprises edge-sharing GaO_6_ chains along the b-axis, which are interconnected by rigid BO_3_ triangles, leaving Pb^2+^ in an asymmetric coordination (see Figure 7). Polycrystalline PbGaBO_4_ was prepared by both conventional and modified solid-state methods. The final calcination temperature was 600 °C for both methods, except that the calcination time for the modified solid-state method was reduced to 5 h instead of 15 h for the conventional solid-state method. The bandgap of the samples obtained by both methods is 4.1 eV, with only a slight difference in the morphology. In terms of photocatalytic performance, the HER of the sample obtained by the modified solid-state method is slightly larger without the addition of the co-catalysts, but the HER of the sample obtained by the conventional solid-state method is slightly larger with a value of 41.9 μmol/h/g after the addition of the dual co-catalysts of RuO_x_ and Pt.

#### 5.2.2. Two-Dimensional Layer Borate Photocatalyst

**Figure 8:**
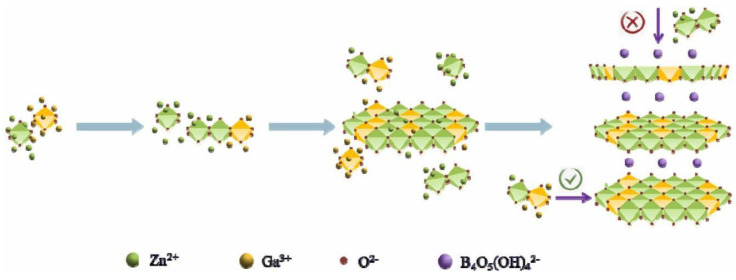
Schematic illustration of the growth mechanism of ZnGa-BLDH [60].

Ultrathin two-dimensional materials, such as Layered Double Hydroxide (LDH), possess characteristics as photocatalysts that include low density, high specific surface area, exposure to more active sites, and shortened charge migration distances. Gu Q. and Liu Z. et al. reported a new borate named ZnGa-BLDH, which was synthesized by the mechanism shown in Figure 8. Metal ions form MO6 octahedral nuclei under alkaline conditions and then gradually form a planar surface by octahedral co-edge connections. Due to the large ionic radius of the intercalated borates, the longitudinal growth between the lamellae was hindered. Nanoparticles were obtained when the reaction system was just heated to 80 °C. With the increasing reaction time, the nanoparticles gradually aggregated to form nanosheet-like structures. After 3 h of reaction, a clear ultra-thin two-dimensional nanosheet structure was formed. The bandgap of ZnGa-BLDH is 4.94 eV. Upon light irradiation, the photogenerated electrons of ZnGa-BLDH itself under UV light and the electrons on the conduction band of ZnGa-BLDH transferred from LUMO of excited CR* are captured by protons to produce hydrogen. The HER of ZnGa-BLDH is 32 μmol/h/g.

#### 5.2.3. Three-Dimensional Network Borate Photocatalyst

Although we already know that the three-dimensional network borate photocatalysts InBO_3_ and Ga-PKU-1 can be used in water splitting reactions, they have also been studied for HEHR and OEHR by Yang T. et al. [61,63]. In previous studies using various methods for the preparation of K_3_Ta_3_B_2_O_12_, it has been shown that the samples from the gel synthesis method have B-deficient defects unfavorable for the separation of photogenerated electron holes, and annealing in oxygen was used to reduce the defects in the preparation of InBO_3_ by Yang T. et al. After annealing treatment, the HER of InBO_3_ was enhanced from 18.2 to 29.5 μmol/h/g. After further loading NiO_x_ on InBO_3_, the HER reached 56.1 μmol/h/g. For Ga-PKU-1, the HER of Pt/Ga-PKU-1 in methanol solution is 323 μmol/h/g, and the OER in silver nitrate solution is 2030 μmol/h/g. The fact that Ga-PKU-1 can be used in both HEHR and OEHR suggests that it has the potential to complete the water splitting reaction from an experimental point of view.

The study by Huppertz H. et al. contributes three borates, In_19_B_34_O_74_(OH)_11_ [64], In_5_B_12_O_25_(OH) [66], and Ga_2_B_3_O_7_(OH) [65], prepared using a high-pressure method, where the harsh high-pressure conditions resulted in all B atoms being B-O tetrahedra, which are interconnected to form an oversized network structure. Supertetrahedra built-up from four tetrahedra can be described as “T2 supertetrahedra” in which the digit 2 stands for the number of tetrahedra that are linked along each edge of the supertetrahedron [73]. In_19_B_34_O_74_(OH)_11_ is the first borate showing the structural motif of a T2 supertetrahedron [54]. Under UV-light irradiation and in pure methanol, the HER of the In_19_B_34_O_74_(OH)_11_ sample is 2.8 μmol/h/g. However, the HER of In_5_B_12_O_25_(OH) is 220 μmol/h/g with the same photocatalytic condition as the In_19_B_34_O_74_(OH)_11_ sample. The differences in photocatalytic performances of hydrogen evolution can be attributed to the differences in the B-O units as well as the differences in the connection between the B-O units and the InO_6_ octahedra. Sample Ga_2_B_3_O_7_(OH), which has the same B-O unit as sample In_5_B_12_O_25_(OH), has an HER of 25.77 μmol/h/g in pure methanol and 9.83 μmol/h/g in aqueous methanol. This is mainly due to the difference between the InO_6_ octahedra and GaO_6_ octahedra with the B-O unit. The comparison of the structural features and photocatalytic performances of these three In/Ga borate photocatalysts fully demonstrates that the B-O units in the borate photocatalysts and the way of connecting the metal-oxygen polyhedra to the B-O units have a significant effect on the photocatalytic performance.

### 5.3. Borate Photocatalysts with Narrow Bandgap (≤3.3 eV) for HEHR or OEHR

Currently, the cations of the borate photocatalysts with narrow bandgap for water decomposition are the first transition metal ions such as Cu^2+^, Co^2+^, and Ni^2+^. Borate photocatalysts can be classified structurally into simple borates and supramolecular borates. The simple borates are CuB_2_O_4_, Cu_3_B_2_O_6_, and Co_3-x_Ni_x_B_2_O_6_ (x = 0, 1, 2, 3) solid solutions. Supramolecular borates are Cu-borate-1 and Cu- borate-2.

**Figure 9:**
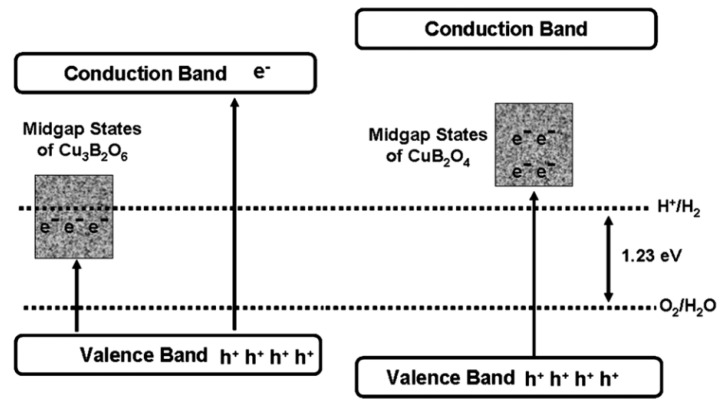
Schematic of the band structures and midgap states of the two copper borates and possible electronic excitations under visible light irradiation [55].

CuB_2_O_4_ and Cu_3_B_2_O_6_ are the same in elemental composition but differ greatly in crystal structure. CuB_2_O_4_ belongs to the tetragonal crystal system, with a three-dimensional mesh of B_3_O_6_ in the B-O unit; Cu_3_B_2_O_6_ belongs to the triclinic crystal system, with an isolated BO_4_ in the B-O unit. The bandgap of CuB_2_O_4_ (3.30 eV) is bigger than that of Cu_3_B_2_O_6_ (2.47 eV) and the former one has better photocatalytic performance in both HEHR and OEHR for hydrogen and oxygen evolution, respectively. Usually, the larger bandgap leads to the weakening of the light-absorbing property, which will make the photocatalytic performance lower. However, this can be explained by the band structure of the two Cu- borates, i.e., the broader bandgap of CuB_2_O_4_ possesses a more negative CB potential and a more positive VB potential relative to Cu_3_B_2_O_6_ (see Figure 9). The HERs of CuB_2_O_4_ and Cu_3_B_3_O_6_ are 69.5 and 14.9 μmol/h/g, under >400 nm visible illumination using methanol solution as the sacrificial agent, and the OERs of CuB_2_O_4_ and Cu_3_B_2_O_6_ were 136.9 and 72 μmol/h/g, under >400 nm illumination using silver nitrate as the sacrificial agent.

Recently, Karadas F. and Ulker E. et al. investigated the photocatalytic performance of oxygen evolution by water decomposition over Co_3-x_Ni_x_B_2_O_6_ solid solution with Na2S2O8 solution as a sacrificial agent under Xe-lamp illumination. The OERs of Co_3_B_2_O_6_, Co_2_NiB_2_O_6_, CoNi_2_B_2_O_6_, and NiB_3_O_6_ were 1822.3, 1020.6, 428.4, and 373.2 μmol/h/g, respectively, which indicated that the photocatalytic performance of oxygen evolution was decreasing with the replacement of Co^2+^ ions by Ni^2+^ ions. The Co_3-x_Ni_x_B_2_O_6_ solid solution belongs to the orthorhombic crystal system and its structural features contain BO_3_ groups and metal-oxygen octahedra. Each metal site is surrounded by an oxygen atom of planar borate groups to afford an octahedral environment. These simple borates show excellent performance in OEHR and they can be used for the design and preparation of Z-scheme photocatalysts.

**Figure 10:**
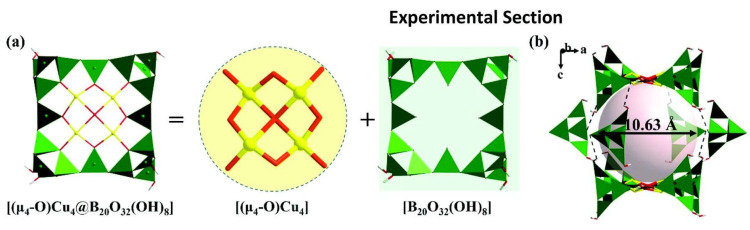
(**a**) View of the porphyrin-shaped [(μ4-O)Cu_4_@B_20_O_40_] cluster; (**b**) view of the large {B_56_} supramolecular cages [59].

Cu-borate-1 and Cu-borate-2 are visible light-responsive photocatalysts with supramolecular cage-like structures that can be used for HEHR. Both copper borates belong to the tetragonal crystal system and possess the same anionic framework structure, so we focus on the description of the crystal structure of Cu-borate-1. The fundamental building block of Cu-borate-1 is the porphyrin-like [(μ4- O)Cu4@B20O32(OH)8] cluster, which is constructed by one [Cu_4_O_9_], four [B_4_O_7_(OH)_2_] clusters and four BO_3_ triangles (Figure 10a). Each {Cu_4_@B_20_} cluster links with another eight {Cu_4_@B_20_} clusters through hydrogen bonds, building the three-dimensional supramolecular framework. Two {Cu_4_@B_20_} and four [B_4_O_7_(OH)_2_] clusters joined together via hydrogen-bond interactions to construct the large {B56} supramolecular cages with a diameter of 10.63 Å (Figure 10b). The HERs of Cu-borate-1 and Cu-borate-2 are ~687.5 and ~562.5 μmol/h/g, respectively.

## 6. Conclusions and Perspectives

This review summarized the application of borate photocatalysts in water decomposition reactions, including total decomposition of water, and water decomposition semi-reactions precipitating hydrogen or oxygen. The main highlight is the attempt to understand the guiding principles for the development of borate photocatalysts starting from the structural analyses of existing borate photocatalysts. The main conclusions obtained in this paper are as follows:

(1) Considering the B-O unit in the borate photocatalyst, the number of B atoms therein is, in principle, less than 6 is more appropriate; the exception is that the number of B atoms in the B-O unit in the borate photocatalyst obtained by the high-pressure method is 12.
(2) From the cationic point of view in borate photocatalysts there are metal-oxygen polyhedra in chains, layers, or networks usually considered to be favorable for photogenerated electron and hole transfer. Therefore, it is also possible to screen borates as photocatalysts from this point of view.
(3) To develop visible light responsive borate photocatalysts, containing the first transition metal borates such as Cu, Co, and Ni can be selected. The photocatalytic oxygen evolution of these first transition metal borates is superior to the photocatalytic hydrogen evolution.
(4) The preparation of borate photocatalysts should in principle avoid high-pressure methods and traditional high-temperature solid-phase methods, even hydrothermal methods, and it is appropriate to choose other soft-chemical synthesis methods, such as sol-gel, precipitation, boric acid flux, and modified solid-state methods. The basic principle is to obtain samples with high specific surface area and low defects.

The challenge for borate photocatalysts for water splitting is primarily the low number of new borate photocatalysts and secondly the difficulty in achieving visible light splitting of water, especially for water splitting reaction. Based on the above conclusions, the development of borate photocatalyst for water decomposition is prospects as follows:

(1) Water decomposition reactions have been an important part of photocatalytic research. The borates as an emerging group of water decomposition photocatalysts, and the conclusions summarised in this paper will contribute to the discovery of new borate photocatalysts.
(2) The first transition metal borates summarised in this paper will help researchers to design new borate-containing composites for water splitting reactions with a visible light-responsive ability.
(3) Improvement of the preparation methods or development of new preparation methods based on the existing synthesis methods of borate photocatalysts is expected.
(4) Knoevenagel condensation of benzaldehyde with malononitrile in the presence of Ga_4_B_2_O_9_ catalyst produces phenylpropanedinitrile with 90% selectivity [70]. Ga_4_B_2_O_9_ efficiently catalyzes the dehydrogenation of n-propanol to obtain malonaldehyde with high selectivity (79%), while Ga-PKU-1 catalyzes the dehydration process to obtain propylene with 94% selectivity [72]. This is attributed to the unique structure of these two borates in generating strong Lewis acids. However, there is a lack of studies on organophotosynthesis with borates.

## Figures and Tables

**Table 1:** Comparison of borate photocatalysts obtained by different synthesis methods using specific surface area and particle size observed by scanning electron microscopy as indicators.

Synthesis Method	Photocatalyst	Synthesis Condition	BET/m^2^g^−1^	SEM/nm	Refs.
Solid-state	K_3_Ta_3_B_2_O_12_	1173 K, 10 h, excess 10% boron	1	300~500	[50,51]
		1173 K, 10 h, excess 20% boron	1	500~800	[50,51]
	InBO_3_	1523 K, 72 h	3	5000~10,000	[52]
	Ga_4_B_2_O_9_	863 K, 10 h	61.7	-	[53]
	Cd_12_Ge_17_B_8_O_58_	1098 K, 15 h	-	~7000	[54]
	CuB_2_O_4_	1073 K, 12 h	negligible	2000	[55]
	Cu_3_B_2_O_6_	1173 K, 36 h	negligible		[55]
	PbGaBO_4_	873 K, 5 h	-	micrometer	[56]
	Ni_a_Co_3−a_B_3_O_4_	1223 K, 24 h	-	-	[49]
Complex gelation	K_3_Ta_3_B_2_O_12_	1073 K, 5 h	1.4	~200	[57]
	Ga_4_B_2_O_9_	893 K, 15 h	33.9	-	[53]
Precipitation	InBO_3_	873 K, 2 h	15	50~200	[52]
		973 K, 2 h or 3 h	14	50~200	[52]
		1023 K, 2 h	10	50~200	[52]
		1073 K, 2 h	5	50~200	[52]
		1273 K, 2 h	1	50~200	[52]
Hydrothermal	Ga_4_B_2_O_9_	513 K, 120 h	3.6	-	[53]
	ZnGa-BLDH	353 K, 12 h	100	5~6	[60]
Boric-acid flux	Ga-PKU-1	493 K, 120 h	3.9	-	[62]
	InBO_3_	513 K, 120 h	13.9~114	20~200	[63]

**Table 3:** Borate photocatalysts for water splitting in pure water under UV-light irradiation.

Photocatalyst	Synthesis Method	Synthesis Condition	BG /eV	Light Source	Mass /mg	Cocatalyst	HER /μmol h^−1^g^−1^	OER /μmol h^−1^g^−1^	AQY (%)	Ref.
K_3_Ta_3_B_2_O_12_	Solid-state	1173 K, 10 h	4.2	450 W-Hg-lamp	1000	-	1790	860		[50]
K_3_Ta_3_B_2_O_12_	Solid-state	1073 K, 20 h	4.2	450 W-Hg-lamp	500	-	4780	2420	6.5@254nm	[50]
K_3_Ta_3_B_2_O_12_	Complex gelation	1073 K, 5 h	4.1–4.2	450 W-Hg-lamp	500	-	210	102	-	[57]
K_3_Ta_3_B_2_O_12_	Complex gelation	1073 K, 5 h	4.1–4.2	450 W-Hg-lamp	500	NiO	784	384	-	[57]
K_3_Ta_3_B_2_O_12_	Solid-state	1073 K, 5 h	4.1	450 W-Hg-lamp	300	-	<50		-	[58]
K_3_Ta_3_B_2_O_12_	Complex gelation	1073 K, 5 h	4.0	450 W-Hg-lamp	300	-	633.3	333.3	-	[58]
K_3_Ta_3_B_2_O_12_	Complex gelation	1073 K, 5 h	4.0	450 W-Hg-lamp	300	NiO	5333.3	-	-	[58]
InBO_3_	Calcite	973 K, 3 h	5.4	450 W-Hg-lamp	500	NiO	796	404	-	[52]
Ga_4_B_2_O_9_	Solid-state	863 K, 10 h	4.12	500 W-Hg-lamp	50	-	118	58	-	[53]
Ga_4_B_2_O_9_	Sol-gel	893 K, 15 h	4.46	500 W-Hg-lamp	50	-	47	22	-	[53]
Ga-PKU-1	Boric-acid flux	493 K, 120 h	4.8	500 W-Hg-lamp	50	RuOx & Pt	28.4	14.5	-	[62]
Cd_12_Ge_17_B_8_O_58_	Solid-state	1098 K, 15 h	4.27	500 W-Hg-lamp	100	NiO_x_	163	-	-	[54]

**Table 4:** Borate photocatalyst for HEHR and OEHR.

Photocatalyst	BG /eV	Light Source	Mass /mg	Sacrificial Agent	Cocatalyst	HER /μmol h^−1^g^−1^	OER /μmol h^−1^g^−1^	Ref.
CuB_2_O_4_	3.30	>400 nm	100	Methanol or AgNO_3_ solution	-	69.5	136.9	[55]
Cu_3_B_2_O_6_	2.47	>400 nm	100	Methanol or AgNO_3_ solution	-	14.9	72	[55]
Cu-Borate-1	2.3	LED-450nm	4	TEOA solution	[Ir(ppy) 2 (dtbbpy)][PF_6_]	~687.5	-	[59]
Cu-Borate-2	2.3	LED-450nm	4	TEOA solution	[Ir(ppy) 2 (dtbbpy)][PF_6_]	~562.5	-	[59]
Co_3_B_2_O_6_	-	DC-Xe-lamp	10	Na_2_S_2_O_8_ solution	[Ru(bpy)_3_]Cl_26_H_2_O	-	1822.3	[49]
Co_2_NiB_2_O_6_	-	DC-Xe-lamp	10	Na_2_S_2_O_8_ solution	[Ru(bpy)_3_]Cl_26_H_2_O	-	1020.6	[49]
CoNi_2_B_2_O_6_	-	DC-Xe-lamp	10	Na_2_S_2_O_8_ solution	[Ru(bpy)_3_]Cl_26_H_2_O	-	428.4	[49]
Ni_3_B_2_O_6_		DC-Xe-lamp	10	Na_2_S_2_O_8_ solution	[Ru(bpy)_3_]Cl_26_H_2_O	-	373.2	[49]
InBO_3_	5.2	500 W-Hg-lamp	500	Methanol solution	-	29.5	-	[63]
InBO_3_	5.2	500 W-Hg-lamp	500	Methanol solution	NiO_x_	56.1	-	[63]
ZnGa-BLDH	4.94	300 W-Xe-lamp	10	TEOA solution		32		[60]
Ga-PKU-1	4.8	500 W-Hg-lamp	100	Methanol or AgNO_3_ solution	Pt	323	2030	[61]
In_19_B_34_O_74_(OH)_11_	-	700 W-Hg-lamp	1.82	Pure methanol	-	2.8	-	[64]
In_5_B_12_O_25_(OH)	-	700 W-Hg-lamp	0.3	Pure methanol	-	220	-	[66]
Ga_2_B_3_O_7_(OH)	4.0	150 W-Hg-lamp	0.18	Pure methanol	-	25.77	-	[65]
Ga_2_B_3_O_7_(OH)	4.0	150 W-Hg-lamp	0.18	Methanol solution	-	9.83	-	[65]
